# Dexamethasone affects human fetal adrenal steroidogenesis and subsequent ACTH response in an *ex vivo* culture model

**DOI:** 10.3389/fendo.2023.1114211

**Published:** 2023-07-06

**Authors:** Cecilie Melau, Berta Gayete Mor, Malene Lundgaard Riis, John E. Nielsen, Eva Dreisler, Kasper Aaboe, Pia Tutein Brenøe, Lea Langhoff Thuesen, Kristine Juul Hare, Rod T. Mitchell, Hanne Frederiksen, Anders Juul, Anne Jørgensen

**Affiliations:** ^1^ Department of Growth and Reproduction, Copenhagen University Hospital, Rigshospitalet, Copenhagen, Denmark; ^2^ International Center for Research and Research Training in Endocrine Disruption of Male Reproduction and Child Health, Copenhagen University Hospital, Rigshospitalet, Copenhagen, Denmark; ^3^ Department of Gynaecology, Copenhagen University Hospital, Rigshospitalet, Copenhagen, Denmark; ^4^ Department of Obstetrics and Gynaecology, Copenhagen University Hospital, Herlev and Gentofte Hospital, Herlev, Denmark; ^5^ Department of Obstetrics and Gynaecology, Copenhagen University Hospital, Hvidovre and Amager Hospital, Hvidovre, Denmark; ^6^ Medical Research Council (MRC) Centre for Reproductive Health, The Queen’s Medical Research Institute, The University of Edinburgh, Edinburgh, United Kingdom; ^7^ Department of Clinical Medicine, University of Copenhagen, Copenhagen, Denmark

**Keywords:** dexamethasone, ACTH-response, human fetal adrenals, *ex vivo* culture, steroidogenesis

## Abstract

**Introduction:**

Administration of dexamethasone (DEX) has been used experimentally to suppress androgenization of external genitalia in 46,XX fetuses with congenital adrenal hyperplasia. Despite this, the prenatal biological mechanism-of-action of DEX on fetal development is not known. This study aimed to examine direct effects of DEX on human fetal adrenal (HFA) steroidogenic activity including possible effects on the subsequent response to ACTH-stimulation.

**Methods:**

Human fetal adrenal (HFA) tissue from 30 fetuses (1^st^ trimester) were cultured *ex vivo* with A) DEX (10 µm) for 14 days, or B) DEX (10 µm) for 10 days followed by ACTH (1 nM) for 4 days. DEX-mediated effects on HFA morphology, viability, and apoptosis (immunohistochemistry), gene expression (quantitative PCR), and steroid hormone secretion (LC-MS/MS) were investigated.

**Results:**

DEX-treatment caused decreased androstenedione (p<0.05) and increased cortisol (p<0.01) secretion suggesting that direct effects on the adrenal gland may contribute to the negative feedback on the hypothalamic-pituitary-adrenal axis *in vivo*. An altered response to ACTH stimulation in HFA pre-treated with DEX included increased androgen (p<0.05) and reduced cortisol production (p<0.05), supporting clinical observations of a temporary decreased ACTH-response following prenatal DEX-treatment. Additionally, the secretion of corticosterone was decreased (p<0.0001) following ACTH-stimulation in the initially DEX-treated HFAs.

**Discussion:**

The observed effects suggest that prenatal DEX-treatment can cause direct effects on HFA steroidogenesis and in the subsequent response to ACTH-stimulation. This may indicate a requirement for careful monitoring of adrenal function in prenatally DEX-treated neonates, with particular focus on their mineralocorticoid levels.

## 1 Introduction

Prenatal treatment of congenital disorders is rare, but one example is maternal administration of the synthetic glucocorticoid dexamethasone (DEX) which has been used experimentally to prevent androgenization of external genitalia in female fetuses affected by congenital adrenal hyperplasia (CAH) (1, 2). Increased levels of adrenal androgens in 46,XX fetuses during fetal sex differentiation (gestational week (GW) ~ 7-14) can have severe consequences since the placental aromatization of androgens to estrogens has not yet been activated. Thus, during the time of sex differentiation the fetoplacental unit cannot protect the fetus against adrenal androgen-driven activation of the androgen receptor in the genital skin (3). To efficiently prevent irreversible virilization of 46,XX fetuses, treatment with DEX must be initiated before genetic diagnosis can be made via a chorionic villous biopsy (at GW 10-12) in order to suppress excess adrenal androgen production in CAH fetuses (4–7). With CAH being an autosomal recessive disorder, the risk of exposing unaffected and/or male fetuses to unnecessary treatment will be seven out of eight, although this risk has been reduced in highly specialized centers where early diagnostic methods to determine fetal sex and diagnosis of classic CAH (around GW 7) have been implemented (8). However, the safety of prenatal DEX treatment continue to be debated since clinical studies have shown conflicting results (9). Recently a survey among thirty-six reference centers of the European Reference Network on Rare Endocrine Conditions (EndoERN) from 14 European countries was conducted. In the 30 centers that responded it was reported that prenatal DEX was currently provided by 36% of the surveyed centers (10). Prenatal DEX treatment has been suspected of causing severe side-effects for both the mother and the fetus, including adverse effects on brain development and behavior (11, 12). Despite this, the safety evaluation of prenatal DEX treatment has been limited by the low number of 46,XX CAH patients and thus a consensus clinical practice guideline from 2018 advocated for the prevention of unnecessary prenatal DEX treatment of CAH fetuses (12).

In contrast to most other glucocorticoids, DEX is not inactivated by placental 11βHSD2 and can therefore reach the fetus. DEX is considered to suppress pituitary adrenocorticotropic hormone (ACTH) production in CAH fetuses (5), which subsequently suppresses the fetal Hypothalamic-Pituitary-Adrenal (HPA) axis and prevents the continually increased secretion of adrenal C_19_ precursors that via both classic and non-classic adrenal steroidogenesis can be converted into androgens and 11-oxygenated androgens (6, 13–18). However, the precise mechanism-of-action of prenatal DEX-treatment is not understood in detail since early human fetal adrenal (HFA) development appears to be independent of ACTH based on observations in anencephalic fetuses (2). Additionally, the exact timing of activation and glucocorticoid feedback sensitivity of the fetal HPA axis remains debated – thus the prenatal mechanism-of-action of DEX treatment could be mediated 1) through suppressed ACTH levels, 2) by an ACTH-independent mode-of-action affecting adrenal HFA steroidogenesis directly, or 3) through combined suppressed ACTH levels and direct effects of DEX on adrenal steroidogenesis.

Currently, the dose of DEX (20 μg/kg maternal body weight) typically used for prenatal treatment of the fetus corresponds to about three to six times supraphysiological for cortisol levels in the mother and may potentially be up to 60 times supraphysiologic for the cortisol levels of a midgestational fetus (12). Since DEX is also a potent agonist for the glucocorticoid receptor (GR), exposure to such supraphysiologic concentrations could activate local organ responses since the GR is expressed in many tissues, including the HFA (19). Despite this, direct treatment effects in HFA tissue have not previously been examined even though clinical observations of neonates (up to 4 months) prenatally exposed to glucocorticoids such as DEX exhibit a blunted response to pain-related stress activation of the HPA axis (20). This observation may suggest a direct effect of DEX treatment on the developing adrenal glands.

This study aimed to explore whether DEX directly affects HFA tissue and determine whether DEX treatment affects a subsequent stimulatory response to ACTH-stimulation. This was examined in our established and extensively validated HFA *ex vivo* tissue culture model, which preserves the viability of HFA cell populations and supports the continued *de novo* biosynthesis of steroid hormones (21, 22). Thus, this study will examine 1) the direct effect of DEX in HFAs *ex vivo* and 2) subsequent ACTH stimulation in DEX treated HFAs in the *ex vivo* culture model.

## 2 Materials and methods

### 2.1 Human fetal adrenal sample collection

Fetal tissue (GW 8-12) used in this study was available following elective surgical termination of pregnancy at Copenhagen University Hospital (Rigshospitalet), Hvidovre Hospital, and Herlev Hospital. Medical staff working independently of the project recruited all participating women who gave their informed written and oral consent. None of the terminations were for reasons of fetal abnormality or pathology of pregnancy. Fetal material was kept at 4°C immediately after termination of pregnancy and during transport to the laboratory. Fetal age was determined by scanning crown-rump length and by evaluation of foot length (23). Fetal adrenal tissue was dissected in ice-cold PBS and immediately setup in *ex vivo* cultures corresponding to a total of 34 intact adrenals from 30 individual fetuses as both adrenals from the same fetus were occasionally used in experimental setup A and B, respectively (including adrenals from 11 male and 19 female fetuses). The collection of and experiments with fetal material used in this study was approved by the regional ethics committee (permit number H-1-2012-007).

### 2.2 *Ex vivo* tissue culture setup

**Figure 1:**
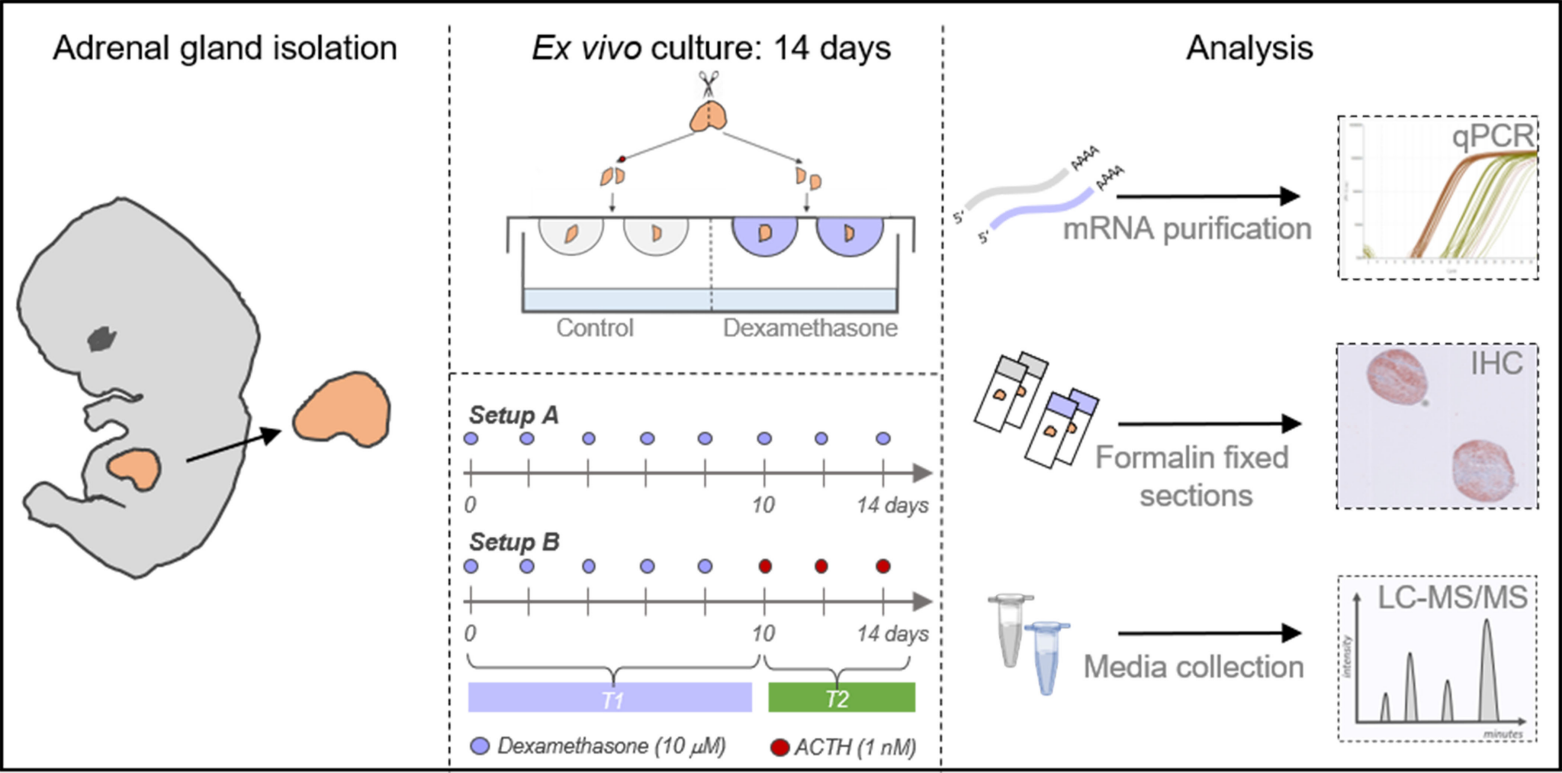
Experimental overview of human fetal adrenal *ex vivo* culture approach. Illustration of the hanging drop culture approach and analysis of cultured tissue and media. Following HFA gland isolation, and dissection into fragments, the tissue pieces were cultured in hanging drops in experimental setup A) where half of the fragments were cultured in vehicle control media and the other half in media containing DEX (10 µM) for 14 days. Alternatively, in experimental setup B) half of the fragments were cultured in vehicle control media and the other half in media with DEX (10 µM) for 10 days followed by 4 days culture of all tissue fragments in media containing ACTH (1 nM). Cultured tissue fragments were either snap frozen and stored at -80°C or fixed in formalin. Transcriptional levels of selected genes were determined using quantitative RT-PCR (qPCR). Protein expression of selected proteins were analyzed by immunohistochemical (IHC) stainings on serial sections. Steroid hormone levels were determined in culture media collected and pooled throughout the experimental period and subsequently measured by LC-MS/MS.

Isolated HFA glands were cultured *ex vivo* as 1 mm^3^ tissue fragments in 40 µl of media at 37°C under 5% CO_2_, in a hanging drop culture approach, as previously described (21, 22). Since each tissue fragment may not have the exact same distribution of cell populations, reproducibility between the different treatment conditions was achieved by initially dividing the adrenal glands into two halves using a dissection microscope (Nikon SMZ800N, Japan) (**Figure 1**) placing the HFA gland on top of Millimeter graph paper. Subsequently, all pieces from one half of the gland, corresponding to 1-11 fragments depending on the initial size of the HFA, were cultured in vehicle-control media (dimethyl sulfoxide (DMSO), 0.1%), and all pieces from the other half of the gland in DEX (10 μM) media. Since a similar HFA cell population is represented by the sum of tissue fragments originating from half an adrenal gland and not the individual tissue fragments, all fragments from one human fetal adrenal gland and treatments group are considered as one biological replicate. The culture media consisted of MEMα media supplemented with 1× MEM non-essential amino acids, 2 mM sodium pyruvate, 2 mM L-glutamine, 1× Insulin, Transferrin and Selenium (ITS) supplement, 1× Penicillin/Streptomycin, 10% Fetal Bovine Serum (FBS). All cell media and supplements were from Gibco (Naerum, Denmark), except ITS (Sigma-Aldrich, Broendby, Denmark).

Treatment effects of DEX was examined in two experimental setups (**Figure 1**). In setup A, HFA fragments were cultured in vehicle control (DMSO) or DEX (10 μM) media for 14 days similar to our previous studies (21, 22). In setup B, the 14-day culture period was divided so that HFA fragments were initially cultured in either vehicle control (DMSO) or DEX (10 μM) media for 10 days followed by 4 days culture in media containing ACTH (1 nM). This set-up was designed to ensure a relatively long period with DEX treatment, while still allowing detection of a possible response to the subsequent ACTH treatment. In both setup A and B, complete media change was performed every 48 hours and the culture media from all replicates from the same sample and treatment were collected and pooled throughout the experimental period to minimize variation from the differences in cellularity between tissue fragments. At the end of the *ex vivo* culture period tissue fragments were either snap frozen and placed at – 80°C until further analysis or cultured in media containing BrdU labeling reagent (Life Technologies, Naerum, Denmark) for 6 hours prior to formalin fixation. DEX (dissolved in DMSO), DMSO, and ACTH (dissolved in H_2_O) were purchased from Sigma-Aldrich (Broendby, Denmark).

### 2.3 Gene expression

Tissue fragments originating from the same sample and treatment group were pooled and snap frozen at the end of the culture period. Total mRNA was extracted from the pooled tissue fragments using the NucleoSpin RNA II purification kit according to the manufacturer’s instructions (Macherey-Nagel, Düren, Germany). The gene expression analysis was conducted as previously described for HFA tissue (24). In brief, cDNA was synthesized using a mixture of dT20 primer and random hexamers (4:1). Real-time polymerase chain reaction (RT-PCR) was performed using specific primers targeting selected genes. All primers were designed to span intron-exon boundaries with optimal annealing temperatures of 62°C, comparable primer length, and CG contents [**Supplementary Table 1**, available in digital research material repository (25)]. Amplicons for *MC2R, StAR, CYP11A1, CYP17A1*, *3βHSD2, CYP21A2, CYP11B1/2* and *SULT2A1* have previously been verified in HFA tissue (24), while amplicons for *SF-1, DAX-1*, and *GR* were initially tested and verified by sequencing (Eurofins MWG GmbH, Ebersberg, Germany). Primer amplification efficiency and detectable dynamic range of all primer sets were validated before analysis. RT-PCR cycle conditions were as follows: one cycle of 3 minutes at 95°C, 40 cycles of 30 seconds at 95°C, 1 minute at 62°C, 1 minute at 72°C, and one cycle of 5 minutes at 72°C. Quantitative RT-PCR analysis was performed in duplicate using Brilliant II SYBR Green qPCR MasterMix (Agilent Technologies, Santa Clara, CA). All experiments were setup in 384-well plates using the Applied Biosystems QuantStudio 5 Real-Time PCR System (Thermo Fisher Scientific) and changes in gene expression quantified using the 2^-ΔΔCt^ method (26). Expression levels were normalized to the reference gene, *RPS20*, and calculated as a ratio relative to the mean of the vehicle controls.

### 2.4 Immunohistochemistry

Immunohistochemistry (IHC) staining was conducted as previously described for formalin fixed samples (21). In brief, paraffin-embedded sections (4 μm) were deparaffinized and rehydrated. Heat-induced antigen retrieval was accomplished in a pressure cooker (medical decloaking chamber, Biocare, Concord, CA, USA) at 110°C for 30 min in citrate buffer (10 mM, pH 6.0). Blocking of endogenous peroxidase was performed with 1% (v/v) H_2_O_2_ in MeOH for 30 minutes followed by incubation in horse serum (20% v/v) with PBS/BSA (5% w/v) (HS) ImmPRESS (Vector Laboratories, Burlingame, California). Tissue sections were washed in Tris-buffered saline between protocol steps and all incubations were carried out in a humidity box. Subsequently, tissue sections were incubated with primary antibodies against BrdU (#M0744, Agilent, RRID: AB_10013660) diluted 1:100, cleaved PARP (#5625, Cell Signaling Technology, RRID: AB_10699459) diluted 1:500 in horse serum overnight at 4°C followed by 1 hour at room temperature. Finally, sections were incubated for 30 minutes with the appropriate ImmPRESS HPR secondary antibody (Vector Laboratories, Burlingame, California, RRID: AB_2336528 and RRID: AB_2336529, respectively) at room temperature. Visualization was performed using ImmPACT AEC peroxidase substrate (Vector Laboratories, Burlingame, California). Sections were counterstained with Mayer’s hematoxylin before mounting with Aquatex (Merck, Darmstadt, Germany). Sections were initially evaluated on a Nikon Microphot-FXA microscope and then by scanning slides on a NanoZoomer 2.0 HT (Hamamatsu Photonics, Herrsching am Ammersee, Germany). Negative controls were included and processed with the primary antibody replaced by the dilution buffer alone, none of which showed staining.

### 2.5 Immunohistochemical quantification

*Ex vivo* cultured tissue viability was evaluated from IHC stainings though determination of BrdU^+^ (proliferation) and poly(ADP-ribose) polymerase cleavage-positive (cPARP^+^, apoptotic) cells per mm^2^ tissue. The entire tissue area of a fragment was quantified and all fragments originating from the same HFA sample and treatment group were analyzed as technical replicates of one biological sample. Tissue area was determined using NDP view software version 2.8.24 (Hamamatsu Photonics, Herrsching am Ammersee, Germany).

### 2.6 LC-MS/MS analysis of culture media

Steroid hormone levels were measured in culture media. The culture media was collected every 48 hours for all tissue fragments originating from the same adrenal sample and treatment group. Media were pooled throughout the 14-day culture period in experimental setup A and throughout day 0-10 (T1) and day 11-14 (T2) in experimental setup B. Steroid hormone levels (nM concentrations) were measured using a method established to quantify steroid metabolites in serum by isotope-dilution TurboFlow-LC-MS/MS, as previously described (27). The method was modified for measurement in culture media (21). This clinically validated analysis package includes the androgens: testosterone, androstenedione, and dehydroepiandrosterone-sulfate (DHEAS); glucocorticoids: cortisone and cortisol; and the steroidogenic intermediates: 11-deoxycortisol, 17-hydroxyprogesterone, progesterone and corticosterone. All measured steroids, except estrone sulfate, are reported in this study. The steroid hormone quantification was performed in three batches, the first in July 2019, the second in July 2020, and the third in November 2021. For all batches, two blanks (water), two un-spiked media controls, two spiked media controls with a mixture of native steroid standards in low concentrations, and two spiked media controls with the native steroid standards in high concentrations were used as method controls, while standards prepared in culture media were used for calibration curves. For several samples, the concentrations of DHEAS, cortisol, and cortisone were out of the standard measurement range and were therefore diluted 1:10 with media and analyzed in a repeated batch. For all analytical batches included in this study, the relative standard deviation (RSD) was < 10% for all steroids in culture media controls spiked in low level, except for DHEAS (< 17%), while RSD was < 4.2% for all steroids in the controls spiked in high level.

### 2.7 Statistics

The effects of DEX treatment were investigated as ratios relative to the mean of vehicle controls. Differences in the percentages of ratios relative to the mean of vehicle controls of immunopositive cells were analyzed using the non-parametric and paired Wilcoxon signed-rank test and graphically illustrated as mean with 95% CI. To meet normality of residuals and homogeneity of variance in the gene expression and steroid hormone analysis, ratios relative to the mean of vehicle controls of gene expression and steroid hormone data from experimental setup A, and ratios relative to T1 hormone levels of the same sample in experimental setup B were transformed by the natural logarithm (ln) and analyzed using paired t-test. Treatment effects were compared with vehicle controls from the same fetus and no outliers were excluded from the dataset. All tests were two-tailed and p<0.05 were considered statistically significant. Treatment effects were back-transformed to obtain the fold change, which was graphically illustrated with data represented as geometric mean with 95% CI. Each sample measurement represents the mean value of all tissue fragments originating from the same sample and treatment group (considered as technical replicates). Statistical analysis and graphical illustrations were performed using GraphPad Prism Software.

## 3 Results

### 3.1 Exposure to dexamethasone does not induce cytotoxic effects in *ex vivo* cultured HFA tissue

**Figure 2:**
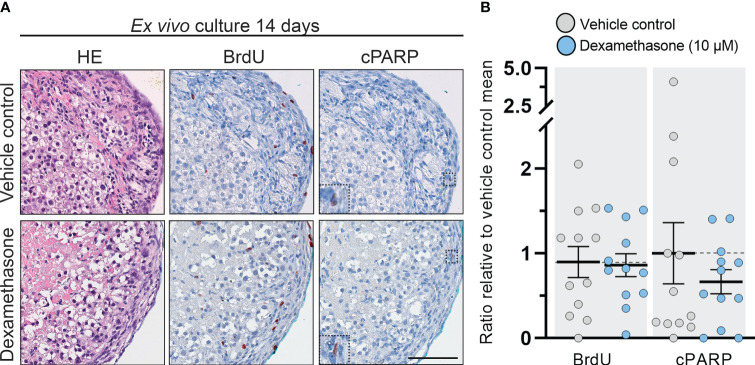
DEX-treatment had no effect on morphology and cell viability in human fetal adrenals cultured *ex vivo*. **(A)** Morphology and expression pattern of BrdU (proliferation marker) and cPARP (apoptosis marker) investigated on serial sections of vehicle control and DEX (10 μM) treated HFA tissue cultured *ex vivo* for 14 days (Experimental setup A). Counterstaining with Mayer’s hematoxylin; scale bar corresponds to 100 μm. **(B)** Quantification of BrdU^+^ and cPARP^+^ cells per mm^2^ tissue relative to the respective mean of vehicle controls. Values represent mean with 95% CI, n = 12 fetuses. The values from the individual fetal samples are shown and represent 1-11 analyzed tissue fragments per fetal sample, depending on the initial size of the adrenal gland. Significant difference between vehicle control and DEX treated samples were analyzed using the Wilcoxon matched-pairs rank test.

No overall changes in morphology, presence of necrotic cores, or apoptosis were observed following treatment with DEX (10 µM) for 14 days in the cultured tissue fragments (**Figure 2A**). Proliferating cells (BrdU^+^) were observed in both the definitive and fetal zones, while only few apoptotic cells (cPARP^+^) were detected in DEX and vehicle control treated samples, thereby suggesting that neither the length of the culture period nor the DEX treatment induced cytotoxic effects. Following quantification, these observations were verified as the number of BrdU^+^ and cPARP^+^ cells/mm^2^ were similar in vehicle controls and DEX treated samples (**Figure 2B**). Together these results suggest that DEX (10 µM) treatment for 2 weeks does not induce cytotoxic effects nor affects proliferation and apoptosis in *ex vivo* cultured HFA tissue.

### 3.2 Dexamethasone treatment does not alter the expression levels of key players in adrenal steroidogenesis

**Figure 3:**
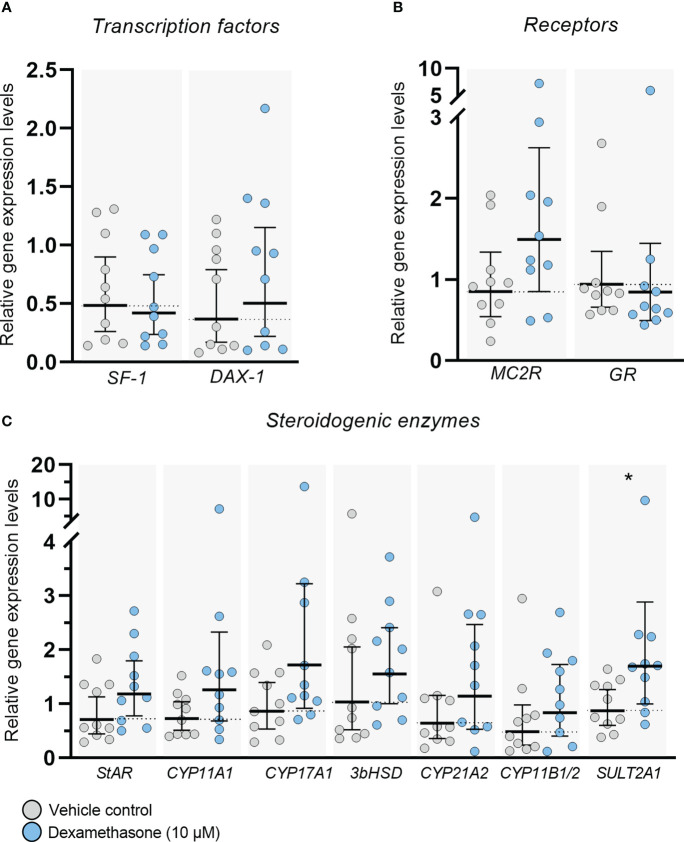
DEX-treatment had no overall effect on gene expression levels in *ex vivo* cultured human fetal adrenals. Gene expression levels of **(A)** transcription factors (*SF-1* and *DAX-1*), **(B)** receptors (*MC2R* and *GR*) and **(C)** enzymes important for adrenal steroidogenesis (*StAR, CYP11A1, CYP17A1*, *3βHSD2, CYP21A2, CYP11B1/2* and *SULT2A1*) examined in *ex vivo* cultured HFA tissue cultured with DEX (10 μM) for 14 days (Experimental setup A). Tissue from 1-11 fragments were pooled per sample and treatment depending on the initial size of half of the adrenal gland and quantitative reverse transcription polymerase chain reaction analysis was performed. Gene expression is relative to the reference gene *RPS20* and normalized to the mean of vehicle controls. Values are shown as geometric mean with 95% CI, n = 10 fetuses. Significant differences between DEX treated and vehicle controls from the same fetus were based on ln-transformed data using paired t-test.

Expression of transcription factors, receptors, and steroidogenic enzymes important for adrenal steroidogenesis were examined in *ex vivo* cultured HFA tissue following treatment with DEX (10 µM) for 14 days (**Figure 3**). No changes in gene expression levels of the nuclear receptor superfamily transcription factors *SF-1* (NR5A1) and *DAX-1* (NR0B1), which are both important regulators of steroidogenesis, were observed at the end of the culture period (**Figure 3A**). Also, no effects of DEX treatment were found on the transcriptional levels of the ACTH receptor, *MC2R*, and the glucocorticoid receptor, *GR* (NR3C1) in *ex vivo* cultured HFA tissue (**Figure 3B**). Importantly, all the classic steroidogenic enzymes examined in this study were expressed in the *ex vivo* cultured tissue regardless of treatment, and no changes in expression level were observed following DEX treatment for 14 days, except the expression of *SULT2A1* which was increased (2.4-fold, *p*<0.05; **Figure 3C**) compared with vehicle controls. The examined steroidogenic enzymes included *StAR, CYP11A1, CYP17A1*, *3βHSD2, CYP21A2, CYP11B1/2* and *SULT2A1* (**Figure 3C**). The expression level of *3βHSD2* was low, but detectable in all examined samples, which is in accordance with previous reports examining 1^st^ trimester (GW 8-12) HFA tissue (24, 28–31). The absolute values of the gene expression levels for examined transcription factors, receptor, and steroidogenic enzymes are shown in **Supplementary Figure 1** [available in digital research material repository (32)], which shows no differences between DEX treated samples and vehicle controls.

### 3.3 Dexamethasone directly affects steroid hormone levels produced in *ex vivo* cultured HFA tissue

**Figure 4:**
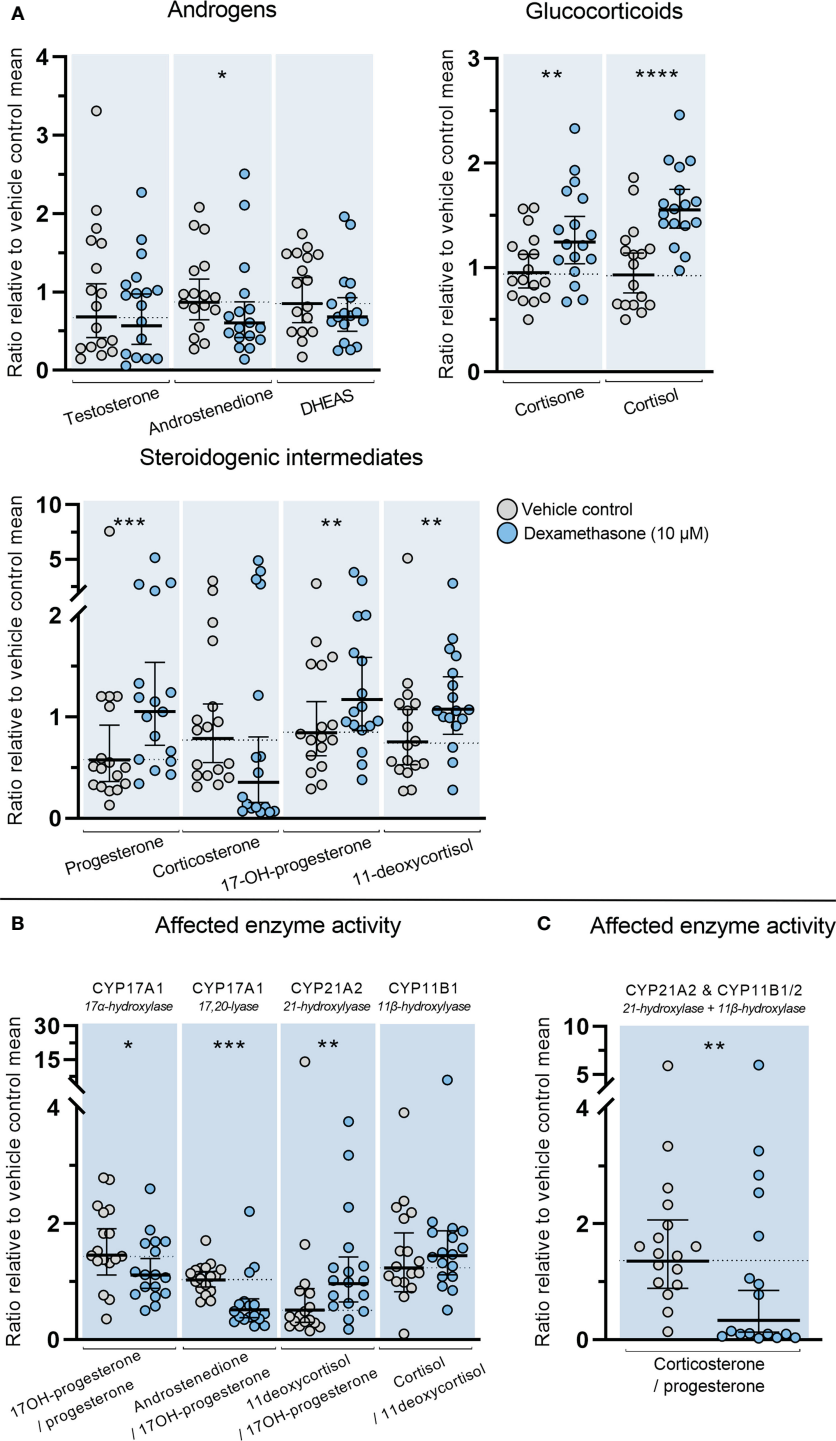
Effects of DEX-treatment on classic steroidogenesis in human fetal adrenals cultured *ex vivo*. Effects of DEX (10 μM) on HFA tissue cultured *ex vivo* for 14 days (Experimental setup A). Secretion of steroid hormones and intermediates was measured by LC-MS/MS in the culture media collected throughout the experimental period. **(A)** Quantification of secreted steroid hormones and intermediate concentrations presented as a ratio relative to the mean of vehicle controls. **(B, C)** Quantification in steroidogenic enzyme activity represented by changes in enzyme product/substrate ratios of **(B)** singular enzymes activities and **(C)** combined enzymes activities. Media was pooled from 1-11 tissue fragments per treatment depending on the initial size of half of the adrenal gland. Values represent geometric mean with 95% CI, n = 17 fetuses. Significant differences of DEX treated samples compared with vehicle controls from the same fetus were based on ln-transformed data using paired t-test. (*) indicate differences compared with vehicle controls, *p<0.05, **p<0.01, ***p<0.001, ****p<0.0001. DHEAS, dehydroepiandrosterone-sulfate.

The direct effects of DEX on classic adrenal steroidogenesis were determined in the *ex vivo* cultured HFA tissue since inhibitory actions through suppression of the HPA axis are not examined in this model. Treatment with DEX (10 µM) for 14 days altered the steroid hormone profile measured in culture media from the *ex vivo* cultured HFA tissue. This included both increased and decreased levels of steroid hormones and steroidogenic intermediates (**Figure 4A**). Treatment with DEX caused a decrease in HFA androstenedione secretion (1.3-fold decrease, *p*<0.05). Interestingly, a direct effect of DEX treatment on the HFA glucocorticoid biosynthesis was also found with increased levels of both cortisone (1.3-fold, *p*<0.01) and cortisol (1.6-fold, *p*<0.0001). Additionally, increased levels of the glucocorticoid intermediates 11-deoxycortisol (1.2-fold, *p*<0.01), and 17-hydroxyprogesterone (1.4-fold, *p*<0.01) were found. Treatment with DEX also increased the level of progesterone (1.4-fold, *p* < 0.001), while there was a tendency towards decreased corticosterone levels although not statistically significant (*p*=0.053).

**Figure 5:**
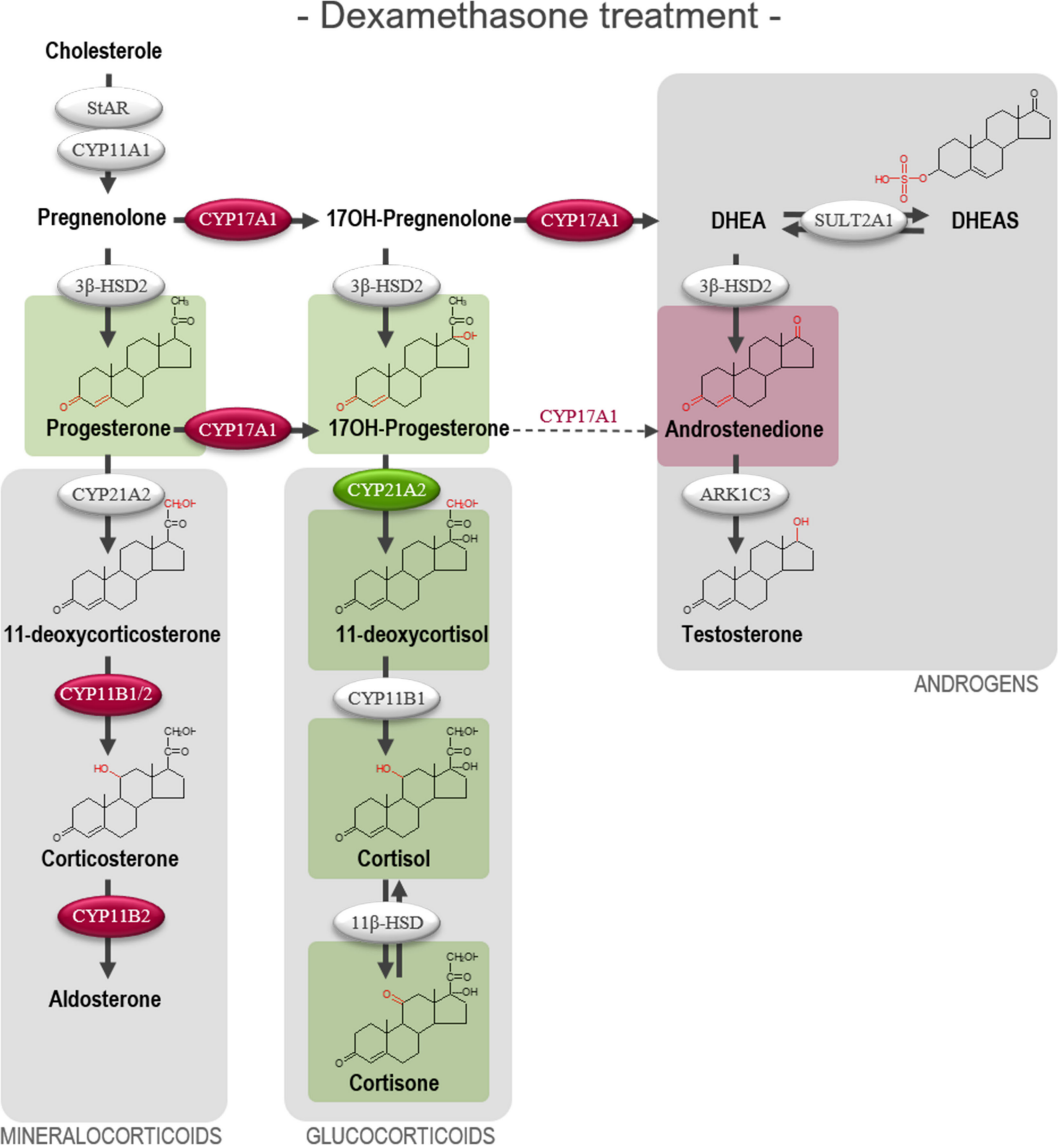
Overview illustrating the affected steroidogenic activity in *ex vivo* cultured human fetal adrenals cultured following DEX-treatment. Schematic overview summarizing the effects of DEX (10 µM) on HFA tissue cultured *ex vivo* for 14 days (Experimental setup A) on classic HFA steroidogenesis. Detailed results are presented in **Figure 4**. Steroid intermediates and hormones measured in this study are shown as chemical structures. Significant changes in secreted steroid levels are indicated by colored boxes. Significantly affected enzyme activities are indicated by colored circles, red: significant decrease, green: significant increase. The dotted arrow indicate reaction with low substrate affinity. DHEA, dehydroepiandrosterone; DHEAS, dehydroepiandrosterone-sulfate.

The altered steroid hormone profile observed in DEX treated HFA tissue was also evident in the product/substrate ratios of several enzymes. Thus, a reduced CYP17A1 enzyme activity resulted in a decreased 17*α*-hydroxylase product/substrate ratio (17-hydroxyprogesterone/progesterone, 1.3-fold decrease, *p*<0.05) and 17,20-lyase product/substrate ratio (androstenedione/17-hydroxyprogesterone, 1.7-fold decrease, *p*<0.001) compared with vehicle controls. Additionally, DEX treatment affected the CYP21A1 activity resulting in an increased 21-hydroxylase product/substrate ratio (11-deoxycortisol/17-hydroxyprogesterone, 0.9-fold, *p*<0.01) but did not affect the CYP11B1 cortisol/11-deoxycortisol ratio (**Figure 4B**). The combined CYP21A2 and CYP11B1/2 product/substrate ratio (corticosterone/progesterone) was also affected (1.5-fold decrease, *p* < 0.01) (**Figure 4C**) which in combination with the results from (**Figure 4B**) suggest an inhibitory effect of DEX on the steroidogenic activity of CYP11B2 (**Figure 4C**). The DEX-mediated changes in HFA steroid hormone profiles and enzyme activity are summarized in **Figure 5**, with adrenal mineralocorticoid, glucocorticoids and classic androgen steroidogenic pathways shown.

### 3.4 Dexamethasone treatment affects the subsequent stimulatory response to ACTH in *ex vivo* cultured HFA tissue

**Figure 6:**
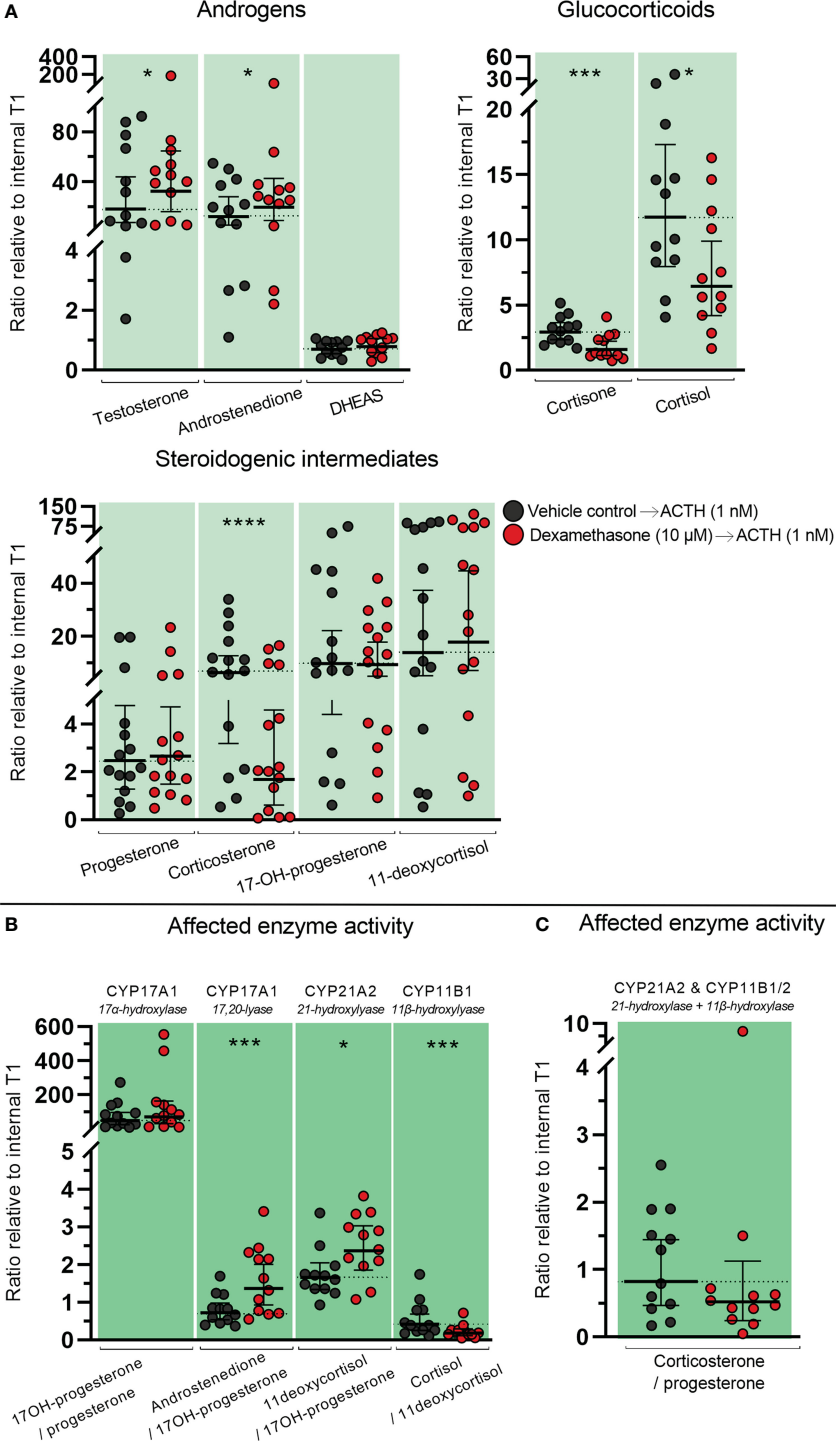
Effects of DEX-treatment on the subsequent stimulatory response to ACTH in human fetal adrenals cultured *ex vivo*. Effects of DEX treatment on a subsequent stimulatory response to ACTH-stimulation in HFA tissue cultured *ex vivo* for 10 days with DEX (10 µM, T1) followed by 4 days with ACTH (1 nM, T2) (Experimental setup B). Secretion of steroid hormones and intermediates was measured by LC-MS/MS in the culture media collected throughout the experimental periods T1 and T2. **(A)** Quantification of secreted steroid hormones and intermediate concentrations presented as differences in internal steroid hormone secretion before and after ACTH-stimulation (T2/T1). **(B, C)** Quantification of steroidogenic enzyme activity represented by changes in enzyme product/substrate ratios of **(B)** singular enzymes activities and **(C)** combined enzymes activities. Media was pooled from 1-11 tissue fragments per treatment depending on the initial size of half of the adrenal gland. Values represent geometric mean with 95% CI, n = 15 fetuses. Significant differences compared with vehicle controls from the same fetus were based on ln-transformed data using paired t-test. (*) indicate differences compared with vehicle controls, *p<0.05, ***p<0.001, ****p<0.0001. DHEAS, dehydroepiandrosterone-sulfate.

Effects of DEX treatment on subsequent stimulatory response to ACTH were examined in *ex vivo* cultured HFA tissue treated with DEX (10 µM) for 10 days (T1) followed by treatment with ACTH (1 nM) for 4 days (T2). The initial treatment with DEX altered the subsequent response to ACTH-stimulation in *ex vivo* cultured HFA tissue compared with vehicle controls. Interestingly the difference in steroid hormone secretion before and after ACTH-stimulation in the same sample (T2/T1, **Figure 6A**) appeared to be opposite to the effects observed from treatment with DEX (10 µM) alone (**Figure 4A**). Specifically, initial DEX-treatment caused an ACTH-mediated increase in both testosterone (1.4-fold, *p*<0.05) and androstenedione secretion (1.5-fold, *p*<0.05) compared with vehicle-control treated samples subsequently stimulated with ACTH (**Figure 6A**). Furthermore, a decrease in cortisone (1.7-fold decrease, *p*<0.001) and cortisol levels (1.8-fold decrease, *p*<0.05) was observed in the samples initially treated with DEX. The ACTH-stimulated secretion of corticosterone was also decreased (2.4-fold, *p*<0.0001) in the samples initially treated with DEX when compared to vehicle control. All other steroidogenic intermediates were secreted in similar levels after ACTH-stimulated in samples initially treated with DEX and vehicle controls.

**Figure 7:**
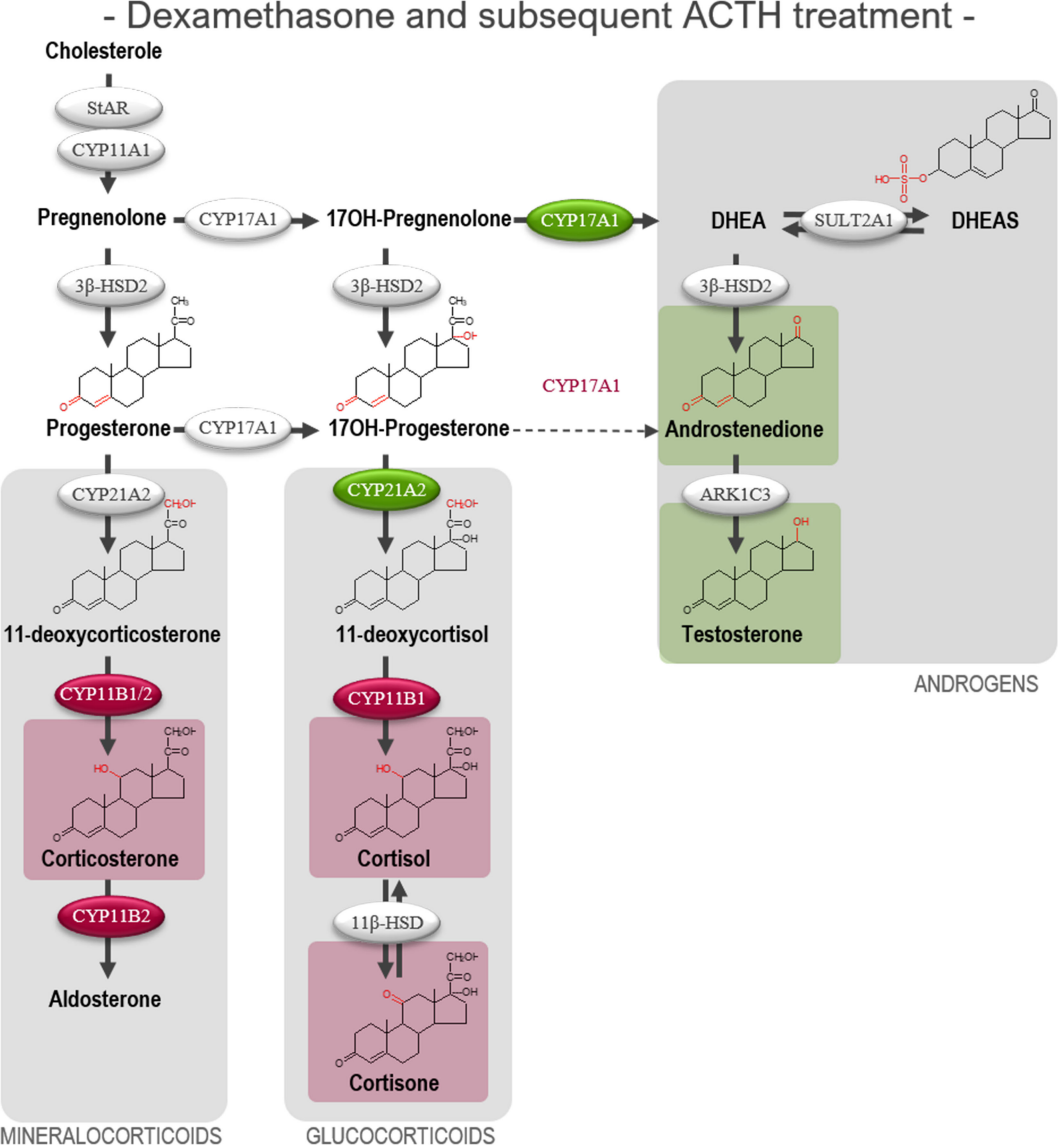
Overview of affected steroidogenic pathways in *ex vivo* cultured human fetal adrenals cultured following DEX-treatment and subsequent ACTH-stimulation. Schematic overview summarizing the effects of treatment with DEX (10 µM) for 10 days followed by ACTH (1 nM) for 4 days on HFA tissue cultured *ex vivo* (Experimental setup B) on classic HFA steroidogenesis. Detailed results are presented in **Figure 6**. Steroid intermediates and hormones measured in this study are shown with chemical structures. Significant changes in secreted steroid levels are indicated by colored boxes. Significantly affected enzyme activities are indicated by colored circles, red: significant decrease, green: significant increase. The dotted arrow indicate reaction with low substrate affinity. DHEA, dehydroepiandrosterone; DHEAS, dehydroepiandrosterone-sulfate.

The initial DEX treatment resulted in increased 17,20-lyase activity of CYP17A1 and CYP21A2 during the subsequent ACTH-stimulation compared with ACTH-stimulated vehicle controls as seen from the affected product/substrate ratios. This was evident from the increased 17,20-lyase product/substrate ratio (androstenedione/17-hydroxyprogesterone, 2.0-fold, *p*<0.001) and 21-hydroxylase product/substrate ratio (11-deoxycortisol/17-hydroxyprogesterone, 1.4-fold, *p*<0.05), while the 17*α*-hydroxylase activity was unaffected (**Figure 6B**). In contrast, initial DEX treatment caused a decrease in the activity of CYB11B1 in the subsequent ACTH-stimulation (cortisol/11-deoxycortisol, 2.4-fold, *p*<0.001) while no effects were observed from the combined CYP21A2 and CYP11B1/2 product/substrate ratio (**Figure 6C**) compared with ACTH-stimulated vehicle controls. The effects of the initial DEX-treatment on the subsequent ACTH-stimulatory response on HFA steroid hormone profiles and enzyme activity are summarized in **Figure 7**, with adrenal mineralocorticoid, glucocorticoids and classic androgen steroidogenic pathways shown.

**Figure 8:**
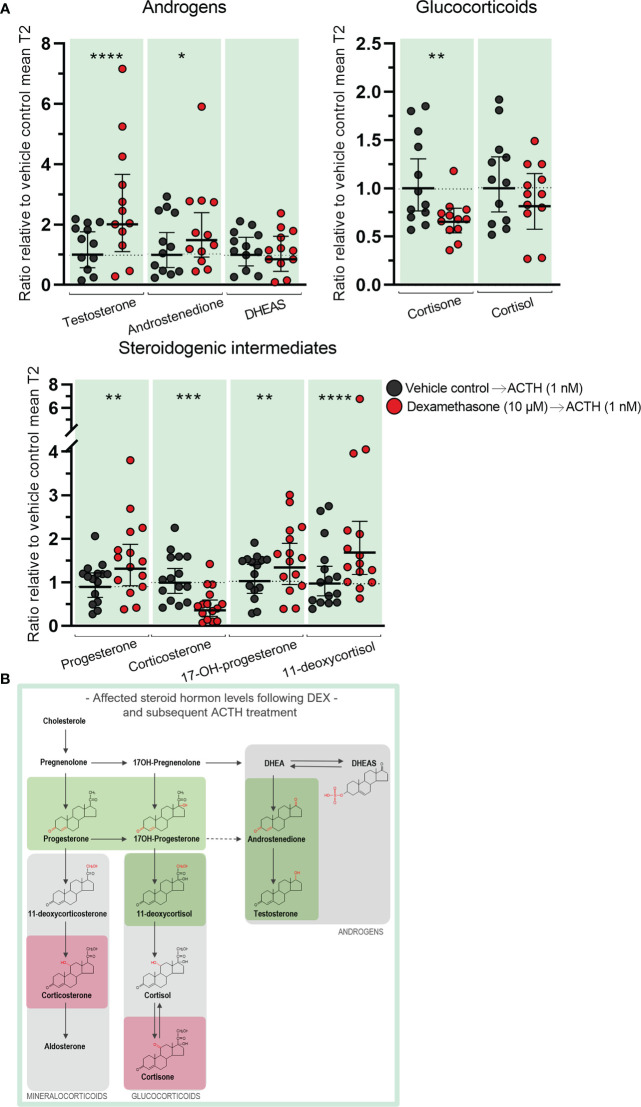
Effects of DEX-treatment on secreted steroid hormone levels when subsequently stimulated with ACTH. Secreted steroid hormones from HFA tissue cultured *ex vivo* for 10 days with DEX (10 µM) were measured after 4 days with subsequent ACTH (1 nM, T2) (Experimental setup B). Secretion of steroid hormones and intermediates was measured by LC-MS/MS in the culture media collected throughout the experimental period T1 and T2. **(A)** Quantification of secreted steroid hormones and intermediate concentrations presented as a ratio relative to the mean of T2 vehicle controls. Media was pooled from 1-11 tissue fragments per treatment depending on the initial size of half of the adrenal gland. Values represent geometric mean with 95% CI, n = 15 fetuses. Significant difference compared with vehicle controls from the same fetus were based on ln-transformed data using paired t-test. (*) indicate differences compared with vehicle controls, *p<0.05, **p<0.01, ***p<0.001, ****p<0.0001. **(B)** Schematic overview summarizing significant effects on secreted steroid hormone levels shown in part A of the figure. Steroid intermediates and hormones measured are shown with chemical structures. Significant changes in secreted steroid levels are indicated by colored boxes, red: significant decrease, green: significant increase. The dotted arrow indicate reaction with low substrate affinity. DHEA, dehydroepiandrosterone; DHEAS, dehydroepiandrosterone-sulfate.

Comparing the difference in steroid hormone secretion before and after ACTH-stimulation in the same sample reflects the effects of DEX on the subsequent response to stimulation (**Figures 6A**, **7**), but it does not illustrate whether the actual secreted steroid levels are different from vehicle controls. When the levels of secreted steroids after ACTH-stimulation (T2 only) are directly compared, it showed increased testosterone (2.1-fold increase, *p*<0.0001) and androstenedione (1.4-fold increase, *p*<0.05) levels in ACTH stimulated DEX treated samples, compared to the levels measured in stimulated vehicle controls, while cortisone levels were reduced (1.6-fold decrease, *p*<0.01) (**Figure 8A**). However, the decreased production of cortisol after ACTH-stimulation in tissue initially treated with DEX (**Figures 6A**, **7**) appeared to compensate for the elevated levels secreted in response to DEX treatment (**Figures 4A**, **5**) resulting in cortisol levels comparable with ACTH-stimulated vehicle controls (**Figure 8A**). Additionally, the initial DEX treatment resulted in increased levels of progesterone (1.4-fold, *p*<0.01), 17-hydroxyprogesterone (1.3-fold, *p*<0.01), and 11-deoxycortisol (1.8-fold, *p*<0.0001), after ACTH-stimulation compared to the levels of vehicle controls (**Figure 8A**). The increased levels of these intermediates reflect the increase observed from the initial DEX treatment (**Figures 4A**, **5**) and not the HFA tissue response to ACTH-stimulation which were unaffected (**Figures 6A**, **7**). Interestingly, the decreased CYP11B1/2 activity following DEX treatment (**Figures 4C**, **5**), the reduced capacity to produce corticosterone in response to stimulation in samples initially treated with DEX (**Figures 6A**, **7**), and the decreased actually secreted corticosterone level (1.9-fold, *p*<0.001) compared with vehicle controls (**Figure 8A**), suggest that corticosterone levels may be particularly affected by DEX-treatment. The effects on the secreted steroid hormone profiles after ACTH-stimulation (T2 only) are summarized in **Figure 8B**, with adrenal mineralocorticoid, glucocorticoids and classic androgen steroidogenic pathways shown.

## 4 Discussion

The effect of prenatal DEX treatment is considered to be via suppression of the fetal HPA axis thereby leading to inhibition of fetal adrenal androgen secretion. However, this assumption requires that the HPA axis and feedback loop is established and functional at this early developmental stage, which is difficult to examine in humans. Since direct treatment effects in HFA tissue have not previously been examined, this study used an established and extensively validated HFA *ex vivo* tissue culture model (21, 22) to examine whether DEX can directly affect healthy HFA tissue and/or whether DEX treatment affects the subsequent stimulatory response to ACTH.

The main finding of the present study was the observed direct effect of DEX treatment on HFA steroidogenesis in the absence of a functional HPA axis using the *ex vivo* culture model. An increased understanding of DEX-mediated effects in healthy HFA tissue is essential since there is a high risk of exposing unaffected fetuses to DEX when there is potential for CAH and prenatal treatment is initiated. Additionally, detailed information about DEX-mediated effects in healthy HFA may provide the basis for extrapolation to treatment effects in tissue from CAH patients. Thus, the information from the present study may be combined with information about enzyme defects to extrapolate or predict effects in CAH foetuses. However, it will not be possible from the findings in the present study to make a direct translation between the effects of the selected treatment concentration used in *ex vivo* culture to an *in vivo* situation.

The treatment of HFA tissue *ex vivo* with DEX (10 µM) did not induce negative effects on HFA tissue morphology or cell viability, and nor was the transcriptional levels of the examined receptors or steroidogenic enzymes affected, except for an increase in *SULT2A1* expression. This is in accordance with results from human adrenocortical NCI-295A cells where no changes in *CYP11A1, CYP17A1* and *3ßHSD2* expression levels were detected following DEX treatment (1-100 µM doses) (2). However, this is in contrast with results from the human adrenocortical NCI-H295R cell line where expression of human adrenal ACTH receptors (*MC2R* and *GR*) was increased by DEX (0.1 µM) (33). Likewise, studies in the same cell line have shown increased expression of *StAR* and *3ßHSD2* following treatment with DEX (0.1 µM) (34). The results from the present study suggest that altered transcriptional levels of adrenal steroidogenic factors are most likely not the main mechanism behind the observed effects of DEX treatment on adrenal steroidogenesis in *ex vivo* cultured HFA tissue.

It was evident from the analysis of steroid hormone levels that DEX treatment can induce direct effects on the adrenal steroidogenesis in *ex vivo* cultured HFA tissue thereby altering the secretion of both classic adrenal androgens, glucocorticoids and steroidogenic intermediates. Since the design of this study excludes DEX-mediated effects via suppression of pituitary ACTH production, the observed direct effects on the HFA tissue contribute to the understanding of the mechanism-of-action of prenatal DEX, which has not previously been examined in detail due to lack of a suitable model system. The expected prenatal DEX-mediated suppression of the fetal HPA axis is supported by a previous study reporting DEX-treatment (1 µM) to suppress the production of ACTH in *ex vivo* cultured human fetal pituitary tissue (GW 10) by 50% (29). However, the precise initiation of fetal pituitary ACTH production is not currently established. Immunohistochemical detection of ACTH has been observed in pituitary tissue from late GW 9 with increased expression observed at GW 10, but *de novo* production of human fetal pituitary ACTH was not examined (29). The observed DEX-mediated decrease in androgen secretion in *ex vivo* cultured HFA in the present study appears to be caused by an inhibitory effect on CYP17A1. This direct DEX-mediated decrease in adrenal androgen secretion may explain how DEX can suppress the virilization of 46,XX CAH fetuses during GW 8-9 where the activity of the fetal HPA-axis may not yet be activated and possibly explain why prenatal DEX-treatment is effective when administrated before GW 9 (35).

Androstenedione, a precursor for 11-oxygenated androgens was reduced following DEX-treatment in the *ex vivo* cultured HFA in the present study. Androstenedione is known to contribute to the excess levels of adrenal androgens in adult CAH patients (36, 37). While *de novo* biosynthesis of 11-oxygenated androgens has not yet been examined in HFA tissue, the CYP11B1 and ARK1C3 enzymes involved in the 11-oxygenated pathways are expressed (24). Thus, it can be speculated that 11-oxygenated androgens may contribute to the virilized phenotype in 46,XX CAH fetuses and that DEX-mediated suppression of 11-oxygenated androgens through the inhibitory effect on CYP11B1 could protect against adrenal androgenization of external genitalia. However, the activity (if any) of the non-classic steroidogenic pathways during fetal life are still unknown and confirmation that HFAs produce *de novo* androgens through the 11-oxygenated pathway remains to be elucidated.

It has previously been speculated that glucocorticoids such as DEX could induce autocrine stimulation of adrenal cortisol production. However, results have been conflicting and depended on the experimental system used. The elevated CYP21A2 activity and increased levels of cortisone, cortisol, and glucocorticoid intermediates in the *ex vivo* cultured HFA tissue in this study supports a stimulating effect of DEX on glucocorticoid production, as described in *in vitro* studies using primary HFA cells (38) and human adrenocortical NCI-H295R cell lines (33, 34). In contrast, a study in the human adrenocortical NCI-H295A cell line examining effects of DEX (up to 100 µM) did not report changes in cortisol production (2) although this may be the result of different steroidogenic profiles of the NCI-H295 cell lines (39). Despite previous studies examining DEX-mediated effects on human adrenal cortisol production *in vitro* it is currently not clear whether mineralocorticoid levels are also affected. This study reports a tendency towards a DEX-mediated decrease in corticosterone levels in HFA cultured tissue, possibly through inhibition of CYP11B2. Whether such a treatment effect could also influence the development of the fetal adrenal glands and possibly the function of the postnatal adrenal should be examined and subsequently included in the safety evaluation of prenatal DEX-treatment of female CAH fetuses. Although the *ex vivo* tissue used in the culture model preserves HFA cell populations and *de novo* steroidogenesis (21) there are some important limitations related to the experimental approach used in the present study. Difference between tissue allocated to treatment and control groups cannot be completely avoided despite the careful effort made to ensure equivalent representation of HFA tissue in the two halves of adrenal glands allocated to fragments in control and treatment groups. However, a relatively high number of biological replicates were included in this study to diminish the impact of such potential differences. These study limitations warrant a cautious interpretation of the reported findings and in particular direct extrapolations between doses used and findings from *ex vivo* culture to an *in vivo* situation should be made with caution. Additionally, from the present study it is not possible to exclude sex- or age-related differences in the response to DEX-treatment. However, based on the results from our previous studies demonstrating no overall sex- or age-related differences in steroid enzyme expression patterns (examined at both transcriptional and protein level), steroidogenic activity in 1^st^ trimester samples (24) or on the effects of three selected inhibitors (Abiraterone acetate, Osilodrostat, and Efavirenz) on human fetal adrenal steroidogenesis under basal and ACTH-stimulated conditions (22) - the present study was not designed to address whether the effects of DEX-treatment differed between male/female and in an age-specific manner during 1^st^ trimester. Thus, future studies examining direct and indirect effects of prenatal DEX-treatment are needed to determine whether such differences exist.

The altered response to ACTH-stimulation observed in HFA tissue initially treated with DEX, indicates that prolonged DEX-treatment may cause long-term effects on HFA steroidogenic activity. Interestingly, the steroid hormone response to ACTH-stimulation in the initially DEX-treated tissue was opposite to the direct treatment effects of DEX in the HFA tissue, which may suggest an internal feedback mechanism ensuring that HFA steroid production is restored to normal. Thus, ACTH-stimulation after DEX treatment resulted in an increased androgen and decreased glucocorticoid and corticosterone response in the *ex vivo* cultured HFA tissue. The effects on ACTH stimulation in the initially DEX-treated HFAs thus mimic the blunted response to pain-related stress activation of the HPA axis observed in neonates prenatally exposed to glucocorticoids such as DEX (20). Likewise, long-term corticosteroid treatment has been reported to cause temporary adrenal insufficiency for a wide variety of conditions, including temporary adrenal insufficiency in postnatal adrenal glands (which take between 2 weeks to 8 months to recover) in childhood acute lymphoblastic leukemia (ALL) patients treated with DEX (40). Importantly, the reduced secretion of corticosterone following ACTH-stimulation in *ex vivo* cultured HFA initially treated with DEX indicate that monitoring for suppression of adrenal function in neonates treated prenatally with DEX may be important.

In conclusion, the present study demonstrates that DEX-treatment can affect human fetal adrenal steroidogenesis directly through an ACTH-independent mode-of-action. While our *ex vivo* culture approach does not exclude that the DEX-mediated effects are to some extent mediated via inhibition of fetal pituitary ACTH-production during early gestation, it demonstrates direct effects of DEX on fetal adrenal steroidogenesis, which includes decreased androgen and increased cortisol secretion. Additionally, direct DEX-mediated effects on subsequent ACTH-stimulatory responses were observed in HFAs indicating that prolonged effects on adrenal activity may be a consequence. These experimental observations support clinical findings suggesting that DEX-treatment can alter the ACTH-response in adrenals, emphasizing that assessment of prenatal DEX-treatment should include identification of temporary adrenal suppression. Finally, DEX-treatment in *ex vivo* cultured HFA decreased CYP11B1/2 steroidogenic activity and reduced the secretion of the aldosterone precursor corticosterone when subsequently stimulated with ACTH, suggesting that careful monitoring of mineralocorticoid levels in neonates prenatally treated with DEX may also be important.

## Data availability statement

The original contributions presented in the study are included in the article/**Supplementary Material**. Further inquiries can be directed to the corresponding author.

## Ethics statement

The studies involving human participants were reviewed and approved by the regional ethical committee of the Capital Region of Denmark. The patients/participants provided their written informed consent to participate in this study.

## Author contributions

CM and AJØ conceived the study and designed the experiments. CM, BG, ML, JN, and HF performed the experiments. ED, KA, PT, LL, and KJ provided study material. CM, BG, ML, JN, HF, RM, AJU, and AJØ analyzed the data. CM and AJØ wrote the manuscript. All authors contributed to the article and approved the submitted version.
