# Supplementary material for: Dexamethasone affects human fetal adrenal steroidogenesis and subsequent ACTH response in an ex vivo culture model
Source: Front Endocrinol (Lausanne). 2023 Jul 6;14:1114211. doi: 10.3389/fendo.2023.1114211 (PMC10358843; doi:10.3389/fendo.2023.1114211)
Supplement: Supplementary file 1 [file Image_1.pdf]

Supplementary Material

Supplementary Figure 1.

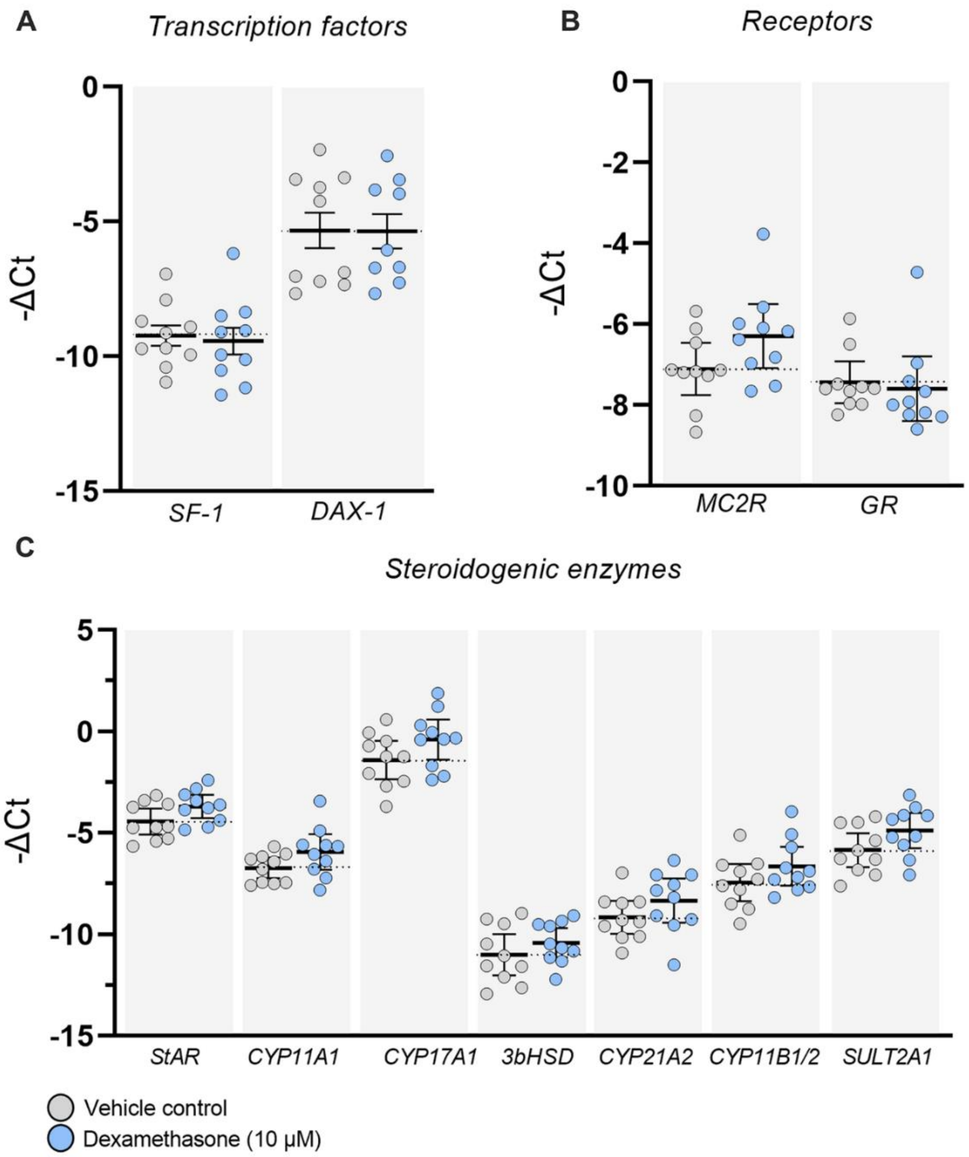

Supplementary Figure 1. Effects of DEX-treatment on gene transcript levels in *ex vivo* cultured human fetal adrenals. Transcript expression levels are shown as $-\Delta Ct$ expression levels ($-\Delta Ct = Ct(Housekeeping\ gene) - Ct(gene\ of\ interest)$) for **A)** transcription factors (*SF-1* and *DAX-1*), **B)** receptors (*MC2R* and *GR*) and **C)** enzymes important for adrenal steroidogenesis (*StAR*, *CYP11A1*, *CYP17A1*, *3βHSD2, CYP21A2, CYP11B1/2* and *SULT2A1*) examined in *ex vivo* cultured HFA tissue cultured with DEX (10 µM) for 14 days (Experimental setup A). Tissue from 1-11 fragments were

pooled per sample and treatment depending on the initial size of half of the adrenal gland and quantitative reverse transcription polymerase chain reaction analysis was performed. Transcript expression is relative to the internal reference gene *RPS20*. Values are shown as mean with 95% CI, n = 10 fetuses. Significant differences between DEX treated and vehicle controls from the same fetus were examined by paired t-test.
