# Supplementary material for: Dexamethasone affects human fetal adrenal steroidogenesis and subsequent ACTH response in an ex vivo culture model
Source: Front Endocrinol (Lausanne). 2023 Jul 6;14:1114211. doi: 10.3389/fendo.2023.1114211 (PMC10358843; doi:10.3389/fendo.2023.1114211)
Supplement: Supplementary file 2 [file Table_1.docx]

Supplementary Material

**Supplementary Table 1.** Primers used for RT-PCR and Quantitative PCR

| ***Gene*** | ***HGNC ID*** | **Forward Primer** | **Reverse Primer** | **Amplicon size (bp)** |
| --- | --- | --- | --- | --- |
| *SF-1* | 12950 | *AAGGCCTGGTGATCCTCAGTCAGC* | *GTGGTAGACATGAGAGACGGTGGAG* | 201 |
| *DAX-1* | 7960 | *CTTGCAGTTCGAGACTGTGGAA* | *GAGGTAGGCGTACTCCTTGGT* | 235 |
| *MC2R* | 6930 | *ACATGGGCTATCTCAAGCCAC* | *TCCAGATGACCGTAAGCACCA* | 204 |
| *GR* | 7978 | *GTCATTCCACCAATTCCCGTTGG* | *GACTCCATAATGACATCCTGAAGC* | 250 |
| *StAR* | 11359 | *CACCCCTAGCACGTGGATTA* | *CTTGGTTGCTAAGGATGCCC* | 152 |
| *CYP11A1* | 2590 | *ATAAACCGACTCCACGTTGC* | *ACAATGGCTGGCTAAACCTG* | 134 |
| *CYP17A1* | 2593 | *GAGTTTGCTGTGGACAAGGG* | *CGCTGGATTCAAGAAACGCT* | 117 |
| *3β-HSD* | 5218 | *CAGGCTCTTTTCAGGAATGG* | *CTTGGACAAGGCCTTCAGAC* | 117 |
| *CYP21A2* | 2600 | *GAGTTCTGTGAGCGCATGAG* | *GAATCACGTCCACAATTTGGAT* | 205 |
| *CYP11B1/2* | 2591/2 | *CTTCCACTACACCATAGAAGCCAGC* | *CCTCAAAGTGCTCCTTCCACAC* | 200 |
| *SULT2A1* | 11458 | *ACAGGACACAGGAAGAACCATAGAG* | *CTTCAGCTTGGGCCACTGTGAA* | 230 |
| *RSP20* | 10405 | *AACAAGCCGCAACGTAAAATC* | *ACGATCCCACGTCTTAGAACC* | 166 |
| *SRY* | 11311 | *GAATATTCCCGCTCTCCGGA* | *GCTGGTGCTCCATTCTTGAG* | 470 |

*All primers are shown in 5’ to 3’ direction. HGNC, HUGO Gene Nomenclature Committee.*
